# Entering the international year of fruits and vegetables: tradeoffs between food production and the environment

**DOI:** 10.1186/s43170-021-00023-0

**Published:** 2021-01-15

**Authors:** Niklaus J. Grünwald

**Affiliations:** Horticultural Crops Research Laboratory, USDA Agricultural Research Services, Corvallis, OR USA

**Fig. 1:**
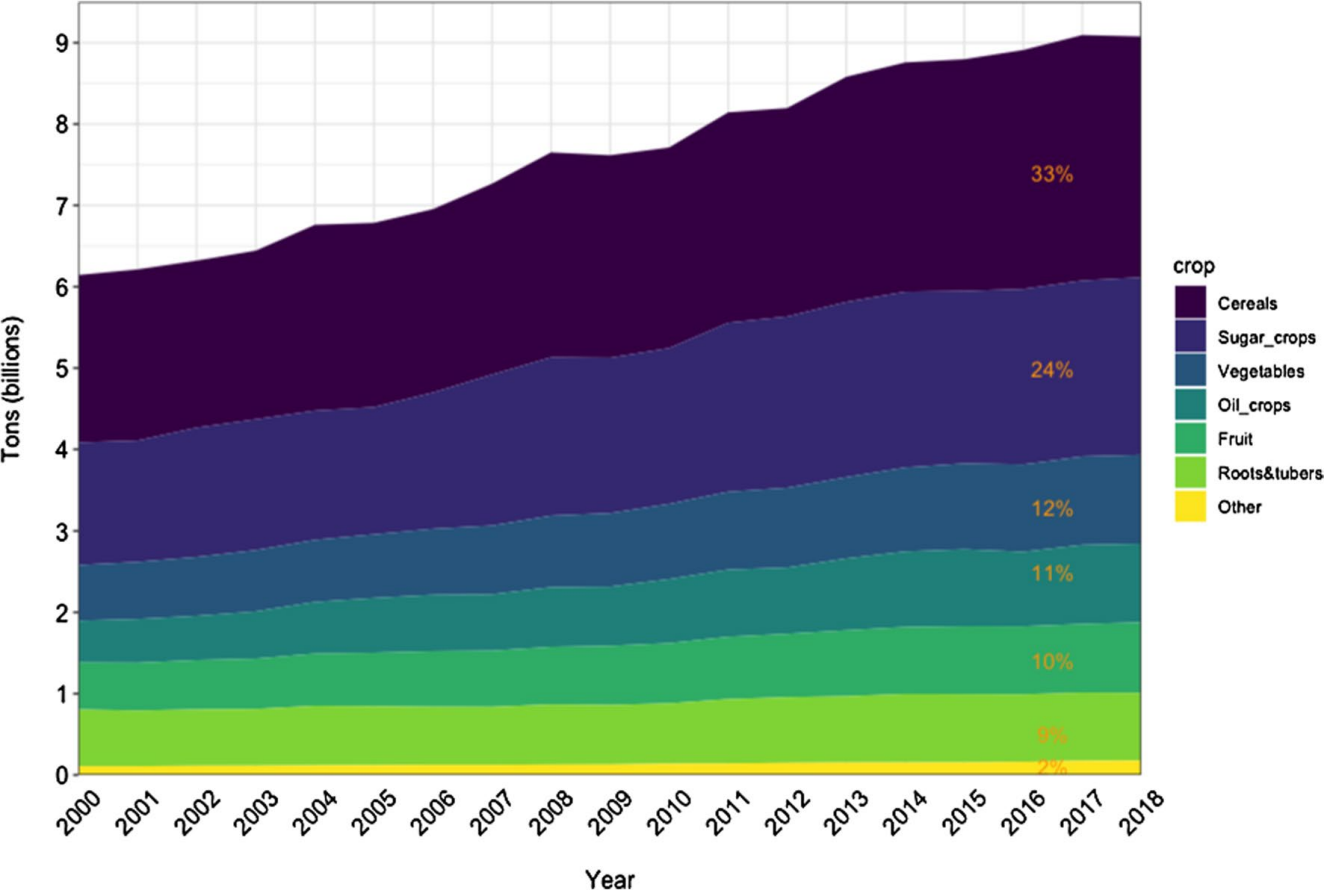
Global crop production in billions of tons by crop. The inset percentages represent the relative percentage of total global production by crop. Source: (World Food and Agriculture—Statistical Yearbook 2020 2020). Fruits and vegetable make up 22% of global crop production combined

As we leave 2020 behind, “the coronavirus lockdown has inspired a surge in gardening not seen since the second world war” (The Economist 2020). While in the Western World COVID-19 inspired gardening, the United Nations have made fruits and vegetables an important component of their messaging. In 2021, we are entering the United Nations’ Year of Fruits and Vegetables with a renewed enthusiasm for gardening. Fruits and vegetable now make up about 22% of food production globally (Fig. 1).

Global food production has increased from 6.1 billion tons in 2000 to 9 billion tons in 2018 (Fig. 1). Our need to feed a growing world population competes with limited resources and provides an environmental impact (Poore and Nemecek 2018). Global food production depletes water resources, degrades global ecosystems, and exacerbates climate change (Foley et al. 2011; Godfray et al. 2010).

*CABI Agriculture and Bioscience* provides a new venue for rapid dissemination of scientific information to address these pressing global problems in agriculture, forestry, and the environments. The journal aims to contribute to solving pressing global issues while facilitating food security. For example, we published a seminal study on the use of antibiotics in low and middle-income countries based on recommendations by agricultural pesticide advisors (Taylor and Reeder 2020). One highlight from this study is that antibiotics are being recommended far more frequently and on a much greater variety of crops than previously thought. Three articles published in *CABI Agriculture and Bioscience* cover the importance of culture collections. Smith et al. (2020) review the importance of mycological culture collections. Close to 800 collections registered with the World Data Centre for Microorganisms hold over three million strains representing a wide range of microbial diversity. The CGIAR banana genebank currently holds 1617 banana accessions from 38 countries as an in vitro collection (Van den Houwe et al. 2020). Van den Houwe et al. (2020) review the importance of safeguarding *Musa* in perpetuity. A research paper by Prasad et al. (2020) studies the diversity of Indian aromatic rice germplasm collections for morphological and agronomical quality traits and molecular characters to identify a core set for crop improvement. Other articles cover aspects of nutrient content of finger millet in Nepal (Luitel et al. 2020), asparagine accumulation in wheat grain (Oddy et al. 2020), spike shedding and stem wilting of pepper in Tanzania (Shango et al. 2020), postharvest storage quality of fresh-cut cactus pears (Kahramanoğlu et al. 2020), temperature control of shoot growth and floral initiation in apple (Heide et al. 2020), and sugar partitioning and metabolism in sweet sorghum (Tovignan et al. 2020). Some further articles focus on plant health such as scab susceptibility of pecan fruit in a native pecan collection in the USA (Bock et al. 2020), novel sources of resistance to apple scab in *Malus* germplasm (Papp et al. 2020), the role of passive surveillance and citizen science in plant health (Brown et al. 2020), the effect of fungal, oomycete and nematode interactions on apple root development in replant soil (Tilston et al. 2020) and the biological control of the South American tomato pinworm using the entomopathogenic fungus *Beauveria bassiana* (Silva et al. 2020). Two studies published by the journal focus on animal health. Sosa et al. show that treatment with colony stimulating factor 2 provides protection to a proportion of blastocysts from cryodamage caused by vitrification (Sosa et al. 2020), whilst Penrith reviews the current status of African swine fever, a serious viral disease of domestic pigs and Eurasian wild boars, which is posing a major threat to pig production (Penrith 2020).

*CABI Agriculture and Bioscience* currently has four concurrent thematic issues in progress including: Disease of tree fruit and nut crops; New approaches to economic impact assessments of non-native pests, pathogens and weeds; Eradication of arthropods: science and society; and Recent advances on sustainable management of arthropod pests in African fruit cropping systems.

The Editorial Board of *CABI Agriculture and Bioscience* has grown to a total of 112 editors, located across all continents, and the journal now includes 19 sections spanning a large range of disciplines, from agroecology to the social sciences. We recently introduced a cohort of Regional Editors-in-Chief currently representing North America, South America, South Asia and Asia Pacific. We will be continuing to recruit further Regional Editors-in-Chief, Section Editors and Associate Editors for our Board in the coming year.
